# Remarks upon Diseases of the Antrum

**Published:** 1884-02

**Authors:** Frank Abbott

**Affiliations:** New York


### REMARKS UPON DISEASES OF THE ANTRUM.

BY FRANK ABBOTT, M. D., NEW YORK.

BEFORE THE DENTAL SOCIETY OF THE STATE OF NEW YORK.

You will excuse me if I talk to you as I would to a class of students, giving you the anatomy of the antrum, its functions, its diseases, their treatment, etc., etc. The antrum of Highmore, or maxillary sinus, is a cavity situated on either side of the nose in the body of the superior maxillary bone. It varies in size from that of a small chestnut to a large hickory nut, in different persons. It is said by some to be an irregular, triangularly-shaped cavity, but I have so far failed to discover one single instance where the shape of an antrum would give me one idea of a triangle. There are some cases where horizontally it will be found to be extremely flat, others very deep; another will be deep from top to bottom, others again very shallow, etc. The floor of the antrum is the alveolar process which is immediately over the ends of the roots of the second bicuspid, first and second molar teeth. The roof of it is the floor of the orbit. The plate between the orbit and the antrum is perhaps one thirty-second of an inch in thickness in its thinest place, but growing thicker as we near the border. The wall separating it from the nares is equally thin. Upon the outside, immediately over the second molar, or between the roots of the first and second molars, the wall is found very thin and easily punctured with a trocar. In the roof of the mouth, on either side, about three-fourths of an inch from the median line, is another point where the wall is thin and easily penetrated, the bone being about one-sixteenth of an inch in thickness. The antrum has two openings into the middle meatus of the nose. One is usually closed by the mucous membrane which lines it. The other is small, only sufficiently large to admit the end of an ordinary probe. When, however, from any cause, the mucous membrane becomes removed, the two openings are readily found, as may be seen in this skull. The same mucous membrane which lines the nares continues through these openings into the antrum, which is lined with it. We have made an opening into it in front, in order that its inner portion may be examined, the thickness of the nasal wall ascertained, etc. Passing an instrument into the antrum through this opening we find that it is in this instance an inch and a quarter in depth, and from the floor of the orbit to the alveolar process (the floor of the antrum) we find it about the same distance, viz., one and one-quarter inches. We have here, therefore, an antrum one and one-fourth inches in two directions. If we now pass the instrument in over the molar teeth, where we also have an opening, we find it coming in contact with the nasal wall at the depth of half an inch. As I said before, it is lined with mucous membrane the same as the nose. Its function is supposed to be to hold a certain amount of air, the same as the frontal sinuses, which are located in the frontal bone above and a little to the right and left of the nose. In this skull they are opened and plainly seen. Not until comparatively recently has it been determined what the function of these sinuses really is. It is supposed that the air here contained, being of the same temperature of the body, mixes with the cold air as it is drawn through the nares on its way to the lungs, and heats it to a certain extent, so that when it reaches the lungs it is more agreeable to them than it otherwise would be, consequently conducive to health. It seems to be a rational explanation at least. You will see very readily, gentlemen, that a severe blow upon the cheek might injure the antrum, as the bony covering is very thin. You can see, also, how a dead tooth may produce disturbance from the protruding of its root through the floor of the antrum. The roots of the first and second molars, and often the second bicuspid, yes, the first bicuspid and canine even, are sometimes involved in an abscess of the antrum. Not long since I had a case under treatment where the lateral incisor had produced an abscess which had worked its way into this sinus. Abscess of the antrum is by far the most frequent disease to which it is subject, and in nearly every case a pulpless tooth is the cause. It is true an abscess may be produced, and is produced by other causes, such as, for instance, a very severe and long-continued coryza, or a severe inflammation of its lining mucous membrane produced by an injury, but such cases are comparatively rare. I presume there is not a person present who has not during a very severe cold in the head, had a feeling of distention well marked in the cheeks and forehead. This is caused by inflammation of the mucous membrane lining the maxillary and frontal sinuses. In such conditions it sometimes happens that the openings from the antrum to the nose become closed, and should the catarrh become chronic, perhaps permanently. Then it is that this sinus becomes filled with an exudation from its own mucous membrane, and unless it be evacuated ulceration will follow, and all the distressing features of an abscess will result. If, however, it is evacuated before ulceration takes place, the case is called dropsy of the antrum. We have cases of this kind where the face swells to an enormous extent, and large lumps in different places are produced upon the face, which eventually discharge. I know of a gentleman who has an opening from the antrum through the cheek, discharging all the time, and the surgeon who has charge of the case is in a fearful quandary to know how to get rid of it. I know of another instance in which the case has been in the hands of a gentleman in this room, where he finds that the opening into the nose has been closed permanently. There has been such a continued inflammation of the mucous membrane in this case, that granulation has taken place, and closed up the naso-antral opening. Until that opening is re-established, he cannot allow the one which he has through the canal, where a tooth came out, to close up. Should he allow it to close, the sinus would fill, and the patient would have all the trials and tribulations attending an abscess of the antrum. We are told by writers upon this subject that it is usually the palatal roots of the molars which penetrate the floor of the antrum. I have examined may skulls for the study of this cavity, its diseases, etc., and in the great majority of them where any roots of the molar teeth were to be found protruding into it, they were the buccal. It is, however, a very easy matter to find any one, and sometimes even all the roots of a molar piercing the floor of this cavity. The reason for this lies in the fact that no two skulls are to be found in which the antrums are just alike. Some are large, some small ; some are located high up, while others are down low ; some are placed forward very prominently, others are to be found very far back. Sometimes in the same skull one antrum is found much higher up than the other, perhaps further back, or much larger. So that in the case of an abscess produced by what is called a dead tooth, it is almost impossible to determine the particular root involved. The first thing to determine is whether such an abscess exists. This leads us to a study of the symptoms, which are, first, a dull, heavy pain of long duration in the cheek of the affected side ; second, tenderness upon pressure over the canine fossa and in the roof of the mouth upon that side; third, a bulging of the wall into the mouth; fourth, an unusual discharge from the nostrils of offensive matter; fifth, a crackling sound caused by the springing of the weakened wall when pressure is produced over the canine fossa. Having determined that an abscess of the antrum exists, we then look for the cause, and, as I have before remarked, we usually find a dead tooth upon that side of the upper jaw, which we at once conclude is the offending organ. This should at once be removed, and an opening into the antrum large enough to admit the point of a syringe, with considerable room to spare, at once effected through the socket nearest the front of the mouth. This is the anterior buccal root socket. I prefer to treat the abscess through this, because it is more convenient to get at. First wash out the antrum thoroughly with warm salt and water, using about a teaspoonful of salt to a glass, or half pint of water. If thrown in with slight force no irritation is produced mechanically, but instead this substance has a very soothing effect upon the inflamed mucous membrane. The large size of the opening through which the point of the syringe is passed admits of the discharge of the excess of material used as a dressing, and being a depending opening it allows the antrum to empty itself, a point always to be observed in the treatment of any disease of this sinus. After using the salt and water, if there is still an offensive odor, syringe with a solution of permanganate of potash and water, two grains to the ounce. This, aside from being the most effective deodorizer we have, is a very good antiseptic. Then syringe with a carbolic solution, one drachm to eight ounces of water, or with Listerine. Then make a plug by winding a bit of cotton around a sprig of broom-corn, first notching it a little so that the cotton will not slip off; dip this into carbolized oil—one part of carbolic acid to fifteen of oil of sweet almonds—then push it into the socket so that it enters the antrum, and in order to secure it tie the end of it which projects into the mouth to an adjoining tooth, or if the teeth are all out and a plate is worn, a notch should be cut in the side of the plate so that it may be fastened to that. These precautions are taken to prevent the plug from being forced into the antrum, or from falling out. This dressing repeat once a day, and if an improvement is observed, make the plug a little smaller each day, letting the opening heal slowly for about ten days, when it will be nearly closed. Then leave the plug out altogether and let it close up. If no improvement is perceptible, however, after two or three dressings, syringe with a solution of iodine, carbolic acid and water. To the carbolic solution I gave you a few minutes since, add an ounce of tincture of iodine. Sometimes I find it necessary to use even a more powerful stimulant, such as the following: Zinc chloride, ten grains to an ounce of water. A saturated solution of chlorate of potash in water sometimes proves very beneficial. After using the above remedies for several days, if there is still a discharge of pus, give the patient sulphide of calcium, one-tenth of a grain pill, three times a day, after meals; this dose may be doubled if necessary. With this treatment I have never failed to get good results.

In case of an abscess, dropsy or any other disease of the antrum, where the teeth are not involved, the best treatment, in my judgment, is to make an opening through the mucous membrane and alveolus between the roots of the first and second molars, being careful that the opening extends to the floor of the antrum, so that by inclining the head slightly it will readily empty itself. Then treat as before described.

Another disease, fortunately seldom met with, is that of polypus. I have never yet seen a case, but should one present itself for my treatment I should, after becoming sure as to diagnosis, proceed to make an opening into the antrum (probably by removing a tooth) large enough to enable me to make a critical examination of its entire wall, and when I had ascertained the point to which the tumor was attached, with a sharp instrument, adapted to the case, detach it, cut or tear it in pieces and remove it. With the probability of a return of the tumor, I should take measures to keep the opening into the antrum open for some months, then, if no new growth was perceptible, allow it to close.

